# The British Hospitals Association: Conference at Birmingham

**Published:** 1912-10-12

**Authors:** 


### THE BRITISH HOSPITALS ASSOCIATION.

### CONFERENCE AT BIRMINGHAM.

### Report of the Proceedings and Discussions (*concluded*).

### The Work and Training of a Hospital Almoner.

By EDGAR S. KEMP, Secretary Hospital Almoners' Council.

The introduction of the subject of this paper at a Conference of the British Hospitals Association does not, I think, call for any apology from me. The growth of the almoner system, first established about twenty years ago at the Royal Free Hospital in London, has been steady; of recent years it has been much accelerated. Two months ago the Report of the Special Committee of King Edward's Hospital Fund for London was issued, and in that Report hospitals are definitely recommended to adopt the system as the first and the most important step to secure the reorganisation of their out-patient departments, the effective treatment of the patients, and the prevention of abuse. The Hospital Almoners' Council, of which I am secretary, is the only body which exists for the purpose of providing training for posts as almoners in the hospitals of London and the provinces.

I want first to try to give some idea of the work of an almoner in a hospital. If I can do this, I think it will be easy for you to appreciate the importance which my Council attaches to the absolute necessity of a thorough and definite training for the work. The almoner lives by co-operation, and her work may be briefly described as the encouragement of the mutual action of the patient and of all who come into contact with him for the purpose of restoring him to health and independence. The almoner is in the hospital as a reminder that doctors and social workers, hospitals and charitable societies, can only do their best work in combination. It is from this point of view, therefore, that she approaches the difficulties which every day present themselves. Her first step will be to ascertain the suitability of the patient for treatment at the hospital. Some may be in a position to pay for their own treatment, and the almoner must know to whom to refer them for the purpose, whether to a provident dispensary or a friendly society to secure regular treatment in trivial illnesses, or to a private doctor. Others may be found to be so poorly off that they are unable to benefit by medical treatment owing to lack of food at home: it may be necessary to refer them to the Guardians, who can supply material as well as medical relief. Two points should be noted in connection with these cases: the inquiries outside the hospital are made not, as a rule, by the almoner personally, but by charitable societies and relief agencies; it should be noted also that no patient is ever turned away by the almoner without the permission of the hospital doctor being obtained. It may be convenient to refer here very briefly to the questions which are often asked with regard to the grounds of the decision as to the suitability of a patient, whether any wage-limit can be fixed, and so on. I think the only reply can be that it is quite impossible to lay down any hard-and-fast rule: some diseases are cheaply dealt with; others require appliances and drugs far beyond the means of even a prosperous working man. Again, a man earning good wages may be contributing largely towards the keep of his parents. Is he to be penalised for that? Particular rules might be laid down for particular classes, when circumstances were normal, but the general principle must always be that each case must be judged on its merits.

When it is decided that the patient may properly be treated at the hospital, it remains for the almoner to see that the treatment is effectively carried out and that the patient is benefiting to the full by it. The most important factor in this matter is the patient himself: I need not labour the point that a cure does not necessarily depend upon the swallowing of a number of bottles of medicine. The patient may not be disposed to follow the doctor's advice as to his mode of life—he may not be able to do so; his home conditions may be bad, his work may be unsuitable—all these are matters with which the almoner must deal. Often the doctor will send the patient to the almoner for some particular purpose, or that she may obtain for him some particular piece of information. It might be thought that the mere provision of convalescent treatment or a surgical instrument would be a simple matter to an almoner accustomed to secure the co-operation of charities outside the hospital. This is true, but it does not necessarily follow that the matter is ended there; instruments must be worn regularly, not on Sundays and other high days and holidays only, as sometimes happens, and they must be properly looked after. Similarly, convalescent treatment may not be the only remedy required. I noticed the other day a reference to the work of one of the almoners at Leeds, in which it was stated that a boy recommended for convalescent treatment by the doctor was found to be living in surroundings so unsuitable that such a course would have been quite ineffective; he was eventually, with the doctor's approval, and in co-operation with three authorities outside the hospital, sent away permanently to a suitable institution where his physical and moral welfare was assured. In many cases no material help is required, advice only will bring about the desired effect; this advice must often be followed up by friendly visiting, which may have to be continued for a prolonged period.

It is important to hospital authorities to note that not only is all the work for the patients outside the hospital done by charitable workers, relief agencies, local nursing associations, district visitors, and others, but that the hospital's Samaritan Fund is only called upon to bear a very small proportion of the cost of material relief. The patients themselves, or their relatives, pay what they can by instalments; their employers may be asked to contribute; the Hospital Sunday Fund is applied to, as well as any charity which may be willing to help. In this way the hospital is saved from becoming a new relief centre in the district. As an example, in the year 1911-1912 surgical instruments were provided for 913 patients at one of the London hospitals through the almoner's department. The money to provide them, about £916, was raised in the following way:—

| | |
|---|---|
| By patients' payments ... ... ... ... | £290 |
| ,, Hospital Sunday Fund grants ... ... | 239 |
| ,, Samaritan Fund ... ... ... ... ... | 139 |
| ,, grants from the C.O.S., clergy, and other sources ... ... ... ... ... ... | 234 |
| ,, outstanding loans, etc. ... ... ... | 14 |
| | £916 |

ARMY MEDICAL LIBRARY WASHINGTON, D. C. U. S. A.

The hospital's share was less than one-seventh of the whole cost.

It will be seen, therefore, how largely the almoner depends upon the good will of those outside the hospital. In return, she owes them the duty of obtaining any information they may require about hospital patients in whom they are interested. She acts as the intermediary between them and the doctor whose advice they require; she saves the hard-worked hospital physician or surgeon, who has no leisure for letter-writing, much time and trouble. Her most important duty in this connection concerns the general practitioner outside the hospital, who would at times like a specialist's opinion on his cases, but who, we are told, often hesitates to send the patient up for fear of losing him. The almoner's duty is to extend, as far as she can, the use of the hospital as a consulting centre. A special point is made of securing advice for those who bring a note from their own doctor with them. As a result, the number of cases sent up for consultative purposes to hospitals with almoners increases every year. In one London hospital the proportion of such cases has risen from 5 per cent. to 26 per cent. of the total number of out-patients.

This necessarily incomplete account of the work of an almoner will be sufficient, I think, to suggest that such duties require an individual carefully selected and trained, and yet it is only of recent years that it has come to be recognised that training is as important for social as well as for any other kind of work. The work of an almoner implies a very considerable interference with the lives of others; how can anyone dare to intervene in so important a matter without being well qualified to do so? The almoner, then, must receive from her training some principle upon which to work; this principle will consist mainly in the application of common sense to her dealings with the hospital patients. She will learn that the greatest asset in a man's troubles is the man himself; that if help be needed it should be given adequately, and so as to secure, as far as possible, against a recurrence of the trouble; that hospitals, like other charities, have their limitations, and that those limitations must be recognised. Apart from this, it is clear that she needs a great deal of purely technical knowledge; it is necessary for her to understand the powers and duties of the public authorities, the Guardians, the Medical Officers of Health, and so on. She must know what charities there are in the place in which she works, what they are prepared to do, and on what terms they will do it. If she works in London she will find that, apart from the local charitable societies in each district, there are special funds for particular classes, as, for instance, soldiers, widows, Scotsmen, and actors. Even the application for a grant from the Hospital Sunday Fund needs to be done in a certain way if it is to be obtained. She must be acquainted with the local lodges and courts of the friendly societies; in the past, almoners have done much to encourage thrift in this direction, in the future they will have to make themselves acquainted with the ramifications of the compulsory thrift enforced by the Insurance Act. The quality which the almoner most needs is tact, not only that she may deal sympathetically and wisely with the patients, but that she may remain on good terms with the secretary, the governing body, and the medical staff of the hospital, as well as with all those others outside the hospital on whom the success of her work so much depends.

The Hospital Almoners' Council, therefore, selects with the greatest care candidates to be trained for almoners' posts. It is recognised that somewhat exceptional qualities are required, and these qualities are, so far as possible, insisted upon. Previous experience of social work, when it has been undertaken on definite lines, is of value, and, in view of the great variety of persons with whom an almoner must deal, a good education and social standing is a distinct asset. Candidates accepted for training are sent for a minimum of six months to different district offices of the Charity Organisation Society in London, where they learn how to write letters, how to interview those in difficulties, and how to get at the root of their troubles; they learn, also, from constant co-operation with the various charities, what is their sphere of operation and method of working. They visit in the homes of the poor, and, when capable, undertake such visits for hospital almoners—the Charity Organisation Society paid nearly 7,000 visits last year for one hospital only. At the same time, they receive instruction at the London School of Sociology, and learn something of the wider view of the case work which they are undertaking daily. When this part of the training is satisfactorily completed, the candidate is sent to a qualified hospital almoner, and learns the actual details of hospital work. This part of the training lasts for another six months at least. In a minimum of a year, therefore, the Council may be prepared to recommend a candidate to a hospital, though, in many cases, she obtains an assistant's post first. There is no examination, and the continuance of a candidate's training depends entirely upon the reports which are regularly received upon her work. I want to emphasise as strongly as I can that the Council claims no control whatever over a candidate after her appointment; she then becomes the servant of the hospital, and is responsible solely to the governing body, which can direct her work as it pleases; it may be added, however, that an almoner trained on the lines I have described and able to profit by the teaching of almoners whose method of working has been gradually built up by experience, should be able, if not unduly hampered, to do work creditable both to the hospital and to herself.

I should like to add a few words on the history and present position of the Hospital Almoners' Council. The system owes its initiation to Mr. C. S. Loch, of the Charity Organisation Society, who has always found time to take the greatest interest in the work of hospitals, and the Society has warmly supported the movement. The Council started work experimentally as an independent body in November 1907, and since that date has trained and given certificates to twenty-one almoners, who are now working in London and the provinces. Nearly a year ago the Council was reconstituted on a fresh basis: its work had proved successful and it was felt desirable that the hospitals themselves should be represented upon it, together with the heads of the principal women's colleges, the almoners themselves and others interested. The Council now numbers about fifty members, including representatives of the governing bodies and medical staffs of many of the London hospitals. Arrangements have recently been made to supply part of the training, in certain cases, in Leeds, where almoners trained by the Council are working. It is hoped later to extend this arrangement to other cities. The lady who is now working at the Birmingham General Hospital was trained by the Council in London.

The future of the movement is, I think, assured: the recognition by the Special Committee of King Edward's Hospital Fund of the value of the system is a striking tribute to the devoted work of those almoners who have paved the way to a better order and a higher standard of treatment in our hospitals. I see only one danger before it, and that is the appointment of almoners unqualified by training or experience: in such cases at the best a modified success for many years is all that can be hoped for; at the worst, utter failure must be expected. No hospital which has employed a qualified almoner has ever put an end to the system: the same cannot be said of those which have appointed untrained workers. It remains for the hospitals to realise that just as they would not permit an unqualified medical man to deal with the ills of the body, so they cannot allow an untrained person to tackle the no less important troubles and difficulties of the patients' lives. The time will come when it will be generally admitted that the more efficient and the more complete method of dealing with patients which the almoner system affords must be adopted for the same reasons that the latest methods of medical treatment are employed in our hospitals. The question will then be, not "Can we afford to have an almoner?" but "Can we afford to do without one?"

### The Discussion.

Dr. Auden, School Medical Officer, Birmingham:—

I believe that no school clinic can be complete without somebody who will carry out the same duties as those which have been described by the reader of the last paper. As you may know, under the Local Education Authorities (Medical Treatment) Act, 1909, local education authorities if they provide school clinics, or treatment for school children, are bound to secure, where it is possible, a sum not exceeding the cost of treatment from the parents of the children so treated, and it is therefore incumbent to make inquiries into the material welfare and social and financial position of the parents seeking treatment for their children. Therefore, I think there is no more useful work than that of a lady almoner in association with our work. In the report which I have made to our local education authority I have urged the appointment of a lady almoner for carrying out work in connection with our dental clinic, because we are proposing to ask for a certain payment, which will be graduated to scale according to the number of children in the family, rent and so on, and unless that is done by somebody thoroughly qualified, who understands the social organisations of the neighbourhood, who is by tact and breeding capable of carrying out those careful investigations, I think that we are liable to cause considerable friction and total failure in getting at the information that we require. There is one point, which is the kernel of the whole question, and that is that adequate remuneration should be given.

Dr. D. J. Mackintosh, Western Infirmary, Glasgow:—

In all our hospitals we have an inquiry officer, who knows the people. I do not see how a lady trained in London for one year is likely to be acquainted with local conditions all over England and Scotland, and be as valuable as an inquiry officer. The almoner is quite a different person. I should like to know where the line is drawn between an almoner and an inquiry officer, or whether they are rolled into one.

Mr. Montague Browne, Northampton:—

I shall be very glad, and I think it would help us very much in the consideration of these matters, if Mr. Kemp could satisfy us that the institution of an almoner does not tend to decrease the voluntary contributions to the hospital, and whether there is not a necessary connection between the employment of an almoner and the question of in-patients' and out-patients' letters. As being a governor for thirty-eight years, my own impression is so adverse to that system of admission by letter that it compels me to the conclusion that the only alternative is to make the hospital in all essentials free, but only subject to investigation.

Mr. J. E. Smith, Children's Hospital, Bristol:—

Can the reader of the paper tell us how many hospitals in the country maintain almoners? I cannot help thinking that the almoner would be much more useful in a very large community than in a small one. In a small town the members of the staff, the members of the committee, those connected with the hospital generally are more or less in touch with most of the people in the town. In Bristol, where I work, we have lately instituted a Civic League. It has really taken the place of the Charity Organisation Society, and I think the officers—and they are mostly voluntary officers—of that Civic League do a great deal of the work suggested for an almoner.

Mr. Kemp, replying upon the discussion:—

Reference has been made to the difference between an almoner and an inquiry officer. The inquiry officer is simply in the hospital as, one might say, a detective pure and simple. An inquiry officer is by no means a substitute for an almoner, and an almoner does through agencies outside the hospital exactly the same kind of work as an inquiry officer. She does it, however, with this additional advantage, that she is not only engaged in that sort of work, but is also primarily engaged in work involving the assistance of the patients, and that makes it far easier for her to take the line of getting rid of patients than if that were her sole duty. With regard to Mr. Montague Browne's question as to the effect of the appointment of an almoner upon subscriptions, I speak mainly from London experience, but some, at any rate, of the London hospitals appeal to the public definitely on the ground that they employ a qualified almoner. I think the almoner should be a strong asset so far as subscriptions are concerned. I do not think an almoner will drive away so many patients that your expenses will be considerably lessened; but if your work is to be done properly you must have one. So far as the almoner's connection with the letter system is concerned, I agree with what has been said as to the evils of that system. At the same time there are hospitals having the letter system, and which also possess an almoner, and except in one explainable instance there have not arisen, so far as I know, any serious difficulties. Mr. Smith, of Bristol, asked how many hospitals have almoners. In London there are twelve or thirteen. Outside there are about six, and an almoner is going to be appointed at a Newcastle hospital next January.

Mr. Howard J. Collins, local Honorary Secretary for the Conference, made a number of announcements, and gave a cordial invitation to any eligible for membership of the Association to join. Personally he believed the Association ought to be of immense value to the work of voluntary hospitals. "Some of us," he added, "are old enough to have had difficulties and messes. Those belonging to the Association believe that they can help others to avoid them. I do hope that we shall not only get more members, but that we shall have more active interest on the part of our members. I hope the work of the British Hospitals Association may go on." (Applause.)

